# Changes in HPV16/18 Prevalence among Unvaccinated Women with Cervical Intraepithelial Neoplasia in Japan: Assessment of Herd Effects following the HPV Vaccination Program

**DOI:** 10.3390/vaccines10020188

**Published:** 2022-01-25

**Authors:** Mamiko Onuki, Kasumi Yamamoto, Hideaki Yahata, Hiroyuki Kanao, Koji Horie, Katsuyuki Konnai, Ai Nio, Kazuhiro Takehara, Shoji Kamiura, Naotake Tsuda, Yuji Takei, Shogo Shigeta, Hidekatsu Nakai, Hiroyuki Yoshida, Takeshi Motohara, Tatsuya Kato, Keiichiro Nakamura, Junzo Hamanishi, Nobutaka Tasaka, Mitsuya Ishikawa, Nobuhiro Kado, Yusuke Taira, Mayuyo Mori, Takashi Iwata, Fumiaki Takahashi, Iwao Kukimoto, Hiroyuki Yoshikawa, Nobuo Yaegashi, Koji Matsumoto, for the MINT Study Group

**Affiliations:** 1 Department of Obstetrics and Gynecology, School of Medicine, Showa University, Tokyo 142-8666, Japan; monuki@med.showa-u.ac.jp; 2 Department of Gynecologic Oncology, Hyogo Cancer Center, Akashi 673-0021, Japan; kasumin_5758@hotmail.com; 3 Department of Gynecology and Obstetrics, Graduate School of Medical Sciences, Kyushu University, Fukuoka 812-8582, Japan; hyahata@med.kyushu-u.ac.jp; 4 Department of Gynecology, Cancer Institute Hospital, Tokyo 135-0063, Japan; hiroyuki.kanao@jfcr.or.jp; 5 Department of Gynecology, Saitama Cancer Center, Saitama 362-0806, Japan; k-horie@saitama-pho.jp; 6 Department of Gynecology, Kanagawa Cancer Center, Yokohama 241-8515, Japan; konnai@kcch.jp; 7 Gynecology Service, NHO Kyushu Cancer Center, Fukuoka 811-1395, Japan; kore1028ai@yahoo.co.jp; 8 Department of Gynecologic Oncology, National Hospital Organization Shikoku Cancer Center, Matsuyama 791-0280, Japan; takehara.kazuhiro.ef@mail.hosp.go.jp; 9 Department of Gynecology, Osaka International Cancer Institute, Osaka 541-8567, Japan; kamiura-sh@oici.jp; 10 Department of Obstetrics and Gynecology, School of Medicine, Kurume University, Kurume 830-0011, Japan; naotakehouston@gmail.com; 11 Department of Obstetrics and Gynecology, Jichi Medical University, Shimotsuke 329-0498, Japan; ytakei@jichi.ac.jp; 12 Department of Obstetrics and Gynecology, Graduate School of Medicine, Tohoku University, Sendai 980-8575, Japan; s.shigeta@med.tohoku.ac.jp (S.S.); yaegashi@med.tohoku.ac.jp (N.Y.); 13 Department of Obstetrics and Gynecology, Faculty of Medicine, Kindai University, Osaka 589-8511, Japan; nakai@med.kindai.ac.jp; 14 Department of Gynecologic Oncology, Saitama Medical University International Medical Center, Saitama 350-1298, Japan; hiro_y@saitama-med.ac.jp; 15 Department of Obstetrics and Gynecology, Faculty of Life Sciences, Kumamoto University, Kumamoto 860-8556, Japan; motorhurry2103@yahoo.co.jp; 16 Department of Obstetrics and Gynecology, Faculty of Medicine, Graduate School of Medicine, Hokkaido University, Sapporo 060-8638, Japan; tatuya-k@med.hokudai.ac.jp; 17 Department of Obstetrics and Gynecology, Dentistry and Pharmaceutical Sciences, Okayama University Graduate School of Medicine, Okayama 700-8558, Japan; k-nakamu@cc.okayama-u.ac.jp; 18 Department of Gynecology and Obstetrics, Graduate School of Medicine, Kyoto University, Kyoto 606-8507, Japan; jnkhmns@kuhp.kyoto-u.ac.jp; 19 Department of Obstetrics and Gynecology, Faculty of Medicine, University of Tsukuba, Tsukuba 305-8575, Japan; tsknbtk@gmail.com (N.T.); hyoshi2626@gmail.com (H.Y.); 20 Department of Gynecology, National Cancer Center Hospital, Tokyo 104-0045, Japan; miishika@ncc.go.jp; 21 Division of Gynecology, Shizuoka Cancer Center Hospital, Shizuoka 411-8777, Japan; n.kado@scchr.jp; 22 Department of Obstetrics and Gynecology, Graduate School of Medicine, University of the Ryukyus, Nishihara 903-0215, Japan; h115474@med.u-ryukyu.ac.jp; 23 Department of Obstetrics and Gynecology, Graduate School of Medicine, The University of Tokyo, Tokyo 113-8655, Japan; mayuyo1976@gmail.com; 24 Department of Obstetrics and Gynecology, School of Medicine, Keio University, Tokyo 160-8582, Japan; iwata@a8.keio.jp; 25 Division of Medical Engineering, Department of Information Science, Iwate Medical University, Yahaba 028-3694, Japan; ftakahas@iwate-med.ac.jp; 26 Pathogen Genomics Center, National Institute of Infectious Diseases, Tokyo 208-0011, Japan; ikuki@nih.go.jp

**Keywords:** adenocarcinoma in situ, cervical cancer, cervical intraepithelial neoplasia, human papillomavirus, vaccination

## Abstract

Since the human papillomavirus (HPV) vaccination program for Japanese girls aged 12–16 years began in 2010, vaccination uptake has been low in women born before 1993 but high (approximately 70%) in those born during 1994–1999. We previously compared the prevalence of vaccine types HPV16 and HPV18 in cervical intraepithelial neoplasia grade 1–3 (CIN1–3) or adenocarcinoma in situ (AIS) between vaccinated and unvaccinated cohorts and found direct protection effects among vaccinated women in Japan. In this study, we focused on changes in HPV16/18 prevalence among “unvaccinated” cohorts with CIN/AIS. We analyzed HPV16/18 prevalence among 5051 unvaccinated women aged <40 years, newly diagnosed with CIN/AIS during 2012–2021 for time trends. Declining trends in HPV16/18 prevalence over 9 years were observed in CIN1 (36.0–10.0%, P_trend_ = 0.03) and CIN2–3/AIS (62.5–36.4%, P_trend_ = 0.07) among women aged <25 years. HPV16/18 prevalence in CIN1 and CIN2–3/AIS diagnosed at age 20–24 years was lower in 1994–1999 birth cohorts compared with 1988–1993 birth cohorts (4.5% vs. 25.7% for CIN1 and 40.0% vs. 58.1% for CIN2–3/AIS, both *p* = 0.04). Significant reduction in HPV16/18 prevalence among young unvaccinated women with CIN1 and CIN2–3/AIS suggests herd effects of HPV vaccination in Japan.

## 1. Introduction

Public funding for human papillomavirus (HPV) vaccination in Japan began for girls aged 12–16 years in 2010. Vaccination uptake is very low (<1%) among women born before 1993 (the “pre-introduction generation”) but high (approximately 40–80%) in women born in 1994–1999 (the “vaccination generation”) [1]. In Japan, a bivalent vaccine, which covers HPV16 and HPV18, was approved in October 2009, and a quadrivalent vaccine, which also targes HPV6 and HPV11, was approved in July 2011. The next-generation 9-valent vaccine, which extends coverage to HPV31, 33, 45, 52, and 58, was licensed in July 2020. The Japanese National Immunization Program includes the bivalent and quadrivalent HPV vaccines, but the 9-valent HPV vaccine is not yet included. The Japanese government withdrew its recommendation for HPV vaccination in June 2013, owing to reports of potential adverse effects after vaccination. Consequently, vaccination coverage among adolescent Japanese women dropped throughout the country, from around 70% in those born during 1994–1999 to only 1% in those born in 2000 or later [1,2]. Suspension of the recommendation for HPV vaccination has continued to the present, despite no scientific or epidemiologic evidence showing a causal link between postvaccination symptoms and HPV vaccination.

As individuals who were vaccinated at the age of 12–16 years between 2010 and 2013 have reached the age of ≥20 years and are recommended for cervical cancer screening; several surveillance studies using cervical screening registries have reported lower incidences of abnormal cytology among young women aged 20–24 years, or those who were vaccinated under the routine immunization program [3,4,5,6,7,8]. Another surveillance study on HPV vaccine effectiveness also reported a lower HPV16/18 infection rate among young, vaccinated cohorts [9].

The impact of HPV vaccination extends beyond the direct protection of vaccinated females. Herd protection, or herd immunity, occurs when a critical number of people have been vaccinated, making it harder for the virus to spread among unvaccinated people. High vaccine coverage is needed to confer herd immunity for unvaccinated women [10]. Herd effects of HPV vaccination have been observed in several countries [10,11,12,13,14]; however, there is no evidence showing indirect protection for unvaccinated women in Japan.

The MINT study is the largest nationwide prospective study monitoring the impact of HPV vaccination and HPV genotype-specific disease incidence in Japan [14,15,16,17]. We examined changes in HPV16/18 prevalence among young women with cervical diseases as the primary endpoint, as a decrease in HPV16/18 prevalence is the earliest indicator of the impact of HPV vaccines. We previously demonstrated a significant reduction in HPV16/18 prevalence among vaccinated women with low- and high-grade cervical lesions in Japan. To evaluate the evidence regarding the herd effects of HPV vaccination in Japan, the current study focused on changes in HPV16/18 prevalence among unvaccinated women diagnosed with cervical abnormalities during 2012–2021.

## 2. Materials and Methods

### 2.1. Study Design

The MINT studies I and II were designed to monitor the long-term population-level impact of HPV vaccination in Japan (the UMIN Clinical Trials Registry: UMIN000008891 and UMIN00038883, respectively). Details of the MINT studies have been described elsewhere [14,15,16,17]. Briefly, our study subjects consist of all women aged 20–39 years (age at registration) newly diagnosed with cervical intraepithelial neoplasia (CIN), adenocarcinoma in situ (AIS) or invasive cervical cancer (ICC). Histological diagnosis was made using HE (hematoxylin and eosin)-stained sections according to the World Health Organization classification. Due to the relevance of clinical practice, we did not review histological specimens used for diagnosis at registration. Women with a previous history of treatment for cervical diseases were excluded. All participants were registered together with their vaccine history. In the MINT study I, a total of 7709 women with cervical abnormalities were registered at 21 participating institutions between August 2012 and December 2017. The ongoing MINT study II uses nearly the same study design and has been in progress since 2019. Because the MINT study II re-started data collection in 2019, monitoring data of 2018 were lacking. In the MINT study II, 1750 women with cervical diseases were recruited at 23 participating institutes between October 2019 and June 2021. Overall, a total of 9459 women with CIN1 (*n* = 870), CIN2–3/AIS (*n* = 7071), or ICC (*n* = 1581) were registered between 2012 and 2021. Both studies rely on self-reported information regarding vaccination status because official vaccination records were not available to determine vaccination status. Serum samples were not collected in the MINT studies I and II. Information on sexual history was obtained using a self-administered questionnaire in the MINT study II, but this information was not collected in the MINT study I.

The study protocol was approved by the institutional review boards of Showa University School of Medicine and participating institutions. Written informed consent was obtained from all patients.

### 2.2. HPV Genotyping Procedures

HPV genotypes in cervical samples were determined using the Linear Array (LA) assay (Roche Molecular Systems, Pleasanton, CA, USA) in the MINT study I and the PGMY-CHUV assay in the MINT study II. Both assays are L1 consensus primer-based PCR methods that use a primer set designated as PGMY09/11 [18]. Details of these HPV genotyping assays are provided elsewhere [19]. Briefly, cervical exfoliated cells were stored in ThinPrep PreservCyt solution (Hologic, Bedford, MA, USA) until DNA extraction. Total cellular DNA was extracted from 200-μL aliquots of cervical exfoliated cells using a QIAamp MinElute Media kit (Qiagen, Valencia, CA, USA) in the MINT study I and a MagNA Pure LC Total Nucleic Acid Isolation kit (Roche) in the MINT study II. PGMY PCR products were subjected to reverse line blot hybridization in both methods.

In the MINT study I, the LA assay was carried out according to the manufacturer’s recommended protocol at an external clinical testing laboratory (SRL, Tokyo, Japan). Briefly, an aliquot (20 μL) of the purified DNA was used for PCR amplification with PGMY09/11 primers. The PCR products were subjected to reverse line blot hybridization for the detection of 37 individual HPV genotypes (HPV6, 11, 16, 18, 26, 31, 33, 35, 39, 40, 42, 45, 51 to 56, 58, 59, 61, 62, 64, 66 to 73, 81 to 84, and 89). LA detects nine HPV genotypes not detected by PGMY-CHUV: HPV61, 62, 64, 67, 71, 72, 81, 82 (IS39) and 89 (CP6108). DNA samples were discarded after the LA assay according to the study protocol.

In the MINT study II, PGMY-CHUV was performed at our laboratory (Pathogen Genomics Center, National Institute of Infectious Diseases, Tokyo, Japan). Briefly, an aliquot (5 μL) of the purified DNA was PCR-amplified (total reaction volume 30 μL) with AmpliTaq Gold polymerase (Thermo Fisher Scientific, Waltham, MA, USA) and biotinylated PGMY09/11 primers to amplify the *L1* gene of mucosal HPVs. Biotinylated human leukocyte antigen (HLA) primers were used to amplify cellular HLA DNA. Positive (0.1 pg/mL of HPV16 full length genomic DNA in a plasmid) and negative controls (dH_2_O) were used to assess the sensitivity of PCR and detect contaminating HPV DNA in reagents. The PCR products (10 μL) were analyzed on 1.5% agarose gels to assess HPV and HLA DNA amplification; amplification of HLA DNA served as an internal control to confirm template integrity. Reverse blotting hybridization was performed as described (Unger et al. 2009). Briefly, 15 μL of denatured PCR products were allowed to hybridize with oligonucleotide probes specific for 31 HPV genotypes (HPV6, 11, 16, 18, 26, 31, 33, 34, 35, 39, 40, 42, 44, 45, 51, 52, 53, 54, 55, 56, 57, 58, 59, 66, 68, 69, 70, 73, 82, 83, and 84) immobilized on a Biodyne C membrane (Pall corporation, Port Washington, NY, USA) using a Miniblotter MN45 (Immunetics, Cambridge, MA, USA). PGMY-CHUV detects three additional HPV genotypes not detected by LA: HPV34, 44 and 57. The hybridized DNA was detected using the horseradish peroxidase-conjugated streptavidin (GE Healthcare, Piscataway, NJ, USA) and the enhanced chemiluminescence detection reagent (GE Healthcare).

The LA and PGMY-CHU assays can detect 28 genotypes in common (HPV6, 11, 16, 18, 26, 31, 33, 35, 39, 40, 42, 45, 51, 52, 53, 54, 55, 56, 58, 59, 66, 68, 69, 70, 73, 82, 83 and 84). All HPV DNA assays were performed by individuals who were blinded to the clinical profile of each patient.

### 2.3. Statistical Methods

Among women reporting no history of HPV vaccination, positive rates for vaccine-types HPV16 or HPV18 in CIN1 and CIN2–3/AIS were analyzed for time trends and according to birth cohort. Data from 2012 through 2020 were analyzed in 2-year periods: 2012–2013, 2014–2015, 2016–2017 and 2019–2020. There were no monitoring data for 2018. Data for 2021 were excluded from the year-on-year trend analyses because only half-year data were available. Age groups were categorized as 20–24, 25–29, 30–34, and 35–39 years. Birth cohorts were analyzed in 3-year periods: 1988–1990, 1991–1993, 1994–1996, and 1997–1999. Fisher’s exact probability test was used for binary comparisons of HPV16/18 positivity. The Cochran–Armitage test was used for time-trend analyses. Linear regression analysis was used to compare year-on-year trends of HPV16/18 prevalence stratified by age (20–24 or 25–39 years) and disease severity (CIN1 or CIN2–3/AIS). The *p*-values obtained in all tests were considered significant at <0.05. We used R version 3.5.1 (R Foundation for Statistical Computing, Vienna, Austria) for the statistical analysis.

## 3. Results

Of the 9459 women registered between August 2012 and June 2021, we obtained HPV genotyping results from 6742 women with CIN1 (*n* = 847; 86 vaccinated [≥1 dose], 754 unvaccinated and 7 unknown), CIN2-3/AIS (*n* = 4549; 169 vaccinated [≥1 dose], 4297 unvaccinated and 83 unknown) and ICC (*n* = 1346; 23 vaccinated [≥1 dose], 1306 unvaccinated and 17 unknown). We could not perform time-trend or birth cohort analyses for ICC due to a very small number of women aged 20–24 years with ICC registered during 2012–2021 (*n* = 20; 2 vaccinated [≥1 dose] and 18 unvaccinated). Thus, the present analyses focused on changes in HPV16/18 prevalence among unvaccinated women with CIN1 and CIN2–3/AIS; their characteristics are summarized in Table 1.

**Figure 1:**
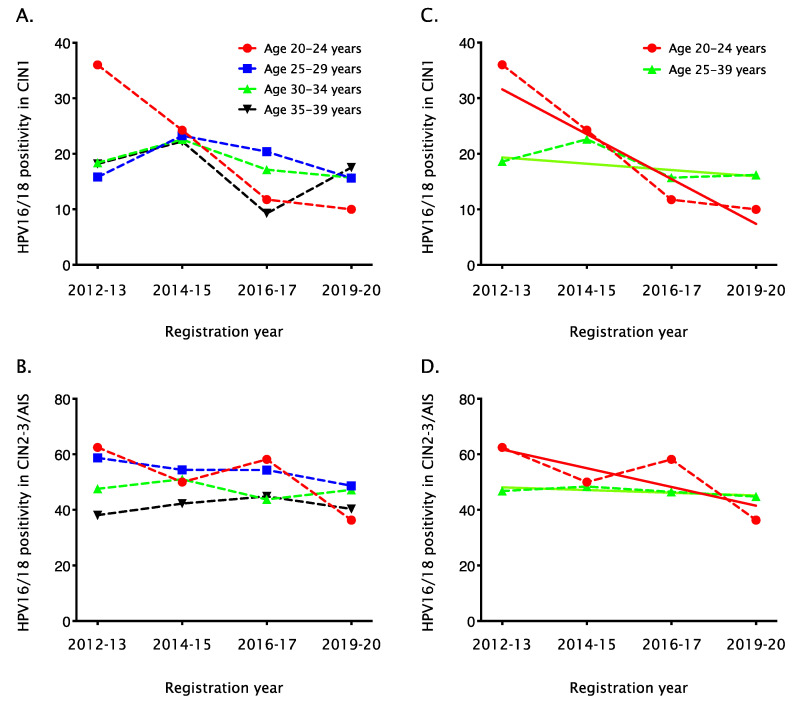
Changes in HPV16/18 prevalence among unvaccinated women with CIN1 or CIN2–3/AIS, by age group. Year-on-year trend of HPV16/18 prevalence among unvaccinated women with CIN1 (**A**) and CIN2–3/AIS (**B**) are shown for four age groups (20–24 years [red], 25–29 years [blue], 30–34 years [green] and 35–39 years [black]). Year-on-year trends of HPV16/18 prevalence (dotted lines) and estimated prevalence trends (solid lines) among unvaccinated women with CIN1 (**C**) and CIN2–3/AIS (**D**) are shown for two age groups (20–24 years [red] and ≥25 years [green]). HPV, human papillomavirus; CIN, cervical intraepithelial neoplasia; AIS, adenocarcinoma in situ.

During the 9-year period of 2012–2020, HPV16/18 prevalence among unvaccinated women aged 20–24 years decreased from 36.0% (9/25) to 10.0% (2/20) in CIN1 (P_trend_ = 0.03, Figure 1A), a decline of 26.0%, and from 62.5% (30/48) to 36.4% (12/33) in CIN2–3/AIS (P_trend_ = 0.07, Figure 1B), a decline of 26.1%. No significant decline was observed in older age groups, although the age group of 25–29 years appeared to be following the age group of 20–24 years.

Using a linear regression model, we compared the linear trends of HPV16/18 prevalence between unvaccinated women aged 20–24 years and those aged ≥25 years. Attribution of HPV16 and HPV18 to CIN1 decreased in unvaccinated women younger than 25 years by 6.9% (95% confidence interval [CI], 4.2–9.6%) per year and in unvaccinated women aged 25 years or older by 1.0% (95% CI, 0.1–2.0%) per year (Figure 1C). Although this result showed a more rapid decline in HPV16/18 prevalence in the age group 20–24 years, the difference in these linear trends did not reach statistical significance (*p* = 0.11). Similarly, HPV16/18 prevalence in CIN2–3/AIS decreased among unvaccinated women younger than 25 years by 5.7% (95% CI, 2.7–8.7%) per year and among unvaccinated women aged ≥25 years by 0.8% (95% CI, 0.2–1.5%) per year (Figure 1D). The declining linear trend of HPV16/18 prevalence was also steeper among unvaccinated women younger than 25 years, but the difference did not reach statistical significance (*p* = 0.19).

**Figure 2:**
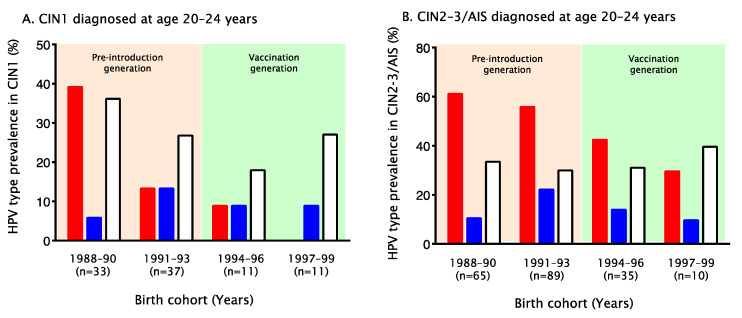
Attribution of HPV16/18, HPV31/33/45 and HPV52/58 to CIN1 and CIN2–3/AIS registered at age 20–24 years, by birth cohort. Even among unvaccinated women, attribution of HPV16/18 (red: ■) to CIN/AIS lesions diagnosed at age 20–24 years was reduced in the 1994–1999 birth cohort (the vaccination generation). Among women reporting no history of HPV vaccination, HPV16/18 prevalence in CIN1 (**A**) registered at age 20–24 years was 39.4% in the 1988–1990 birth cohort (*n* = 33), 13.5% in the 1991–1993 birth cohort (*n* = 37), 9.1% in 1994–1996 birth cohort (*n* = 11), and 0.0% in the 1997–1999 birth cohort (*n* = 11) (P_trend_ = 0.002). Similarly, HPV16/18 prevalence (red) in CIN2–3/AIS (**B**) registered at age 20–24 years was 61.5% in the 1988–1990 birth cohort (*n* = 65), 56.2% in the 1991–1993 birth cohort (*n* = 89), 42.9% in the 1994–1996 birth cohort (*n* = 35), and 30.0% in the 1997–1999 birth cohort (*n* = 10) (P_trend_ = 0.002). For HPV31/33/45 (blue: ■) and HPV52/58 (white: □), however, no significant increase or decrease in prevalence was observed. HPV, human papillomavirus; CIN, cervical intraepithelial neoplasia; AIS, adenocarcinoma in situ.

Next, we analyzed the HPV16/18 prevalence in CIN1 and CIN2–3/AIS among registered women aged 20–24 years according to birth cohort. Among unvaccinated women, HPV16/18 prevalence in CIN1diagnosed at age 20–24 years was 39.4% (13/33) in the 1988–1990 birth cohort (*n* = 33), 13.5% (5/37) in the 1991–1993 birth cohort (*n* = 37), 9.1% (1/11) in the 1994–1996 birth cohort (*n* = 11), and 0.0% (0/11) in the 1997–1999 birth cohort (*n* = 11) (P_trend_ = 0.002, Figure 2A). The HPV16/18 prevalence was also significantly different between the pre-introduction generation (1988–1993 birth cohort) and the vaccination generation (1994–1999 birth cohort) (25.7% [18/70] vs. 4.5% [1/22], *p* = 0.04). Moreover, in the pre-introduction generation, HPV16/18 prevalence was significantly lower in the 1991–1993 birth cohort than in the 1988–1990 birth cohort (*p* = 0.02). HPV16/18 prevalence in CIN1 was only 2.1% among vaccinated women aged 20–24 years (*n* = 48) in the 1988–1999 birth cohorts; we confirmed that attribution of HPV16 and HPV18 to CIN1 differed remarkably between vaccinated and unvaccinated women (2.1% [1/48] vs. 20.7% [19/92], *p* = 0.002).

When the analysis was restricted to unvaccinated women diagnosed with CIN2–3/AIS at 20–24 years, the HVP16/18 prevalence was 61.5% (40/65) in the 1988–1990 birth cohort (*n* = 65), 56.2% (50/89) in the 1991–1993 birth cohort (*n* = 89), 42.9% (15/35) in the 1994–1996 birth cohort (*n* = 35), and 30.0% (3/10) in the 1997–1999 birth cohort (*n* = 10) (P_trend_ = 0.02, Figure 2B). The difference in HPV16/18 prevalence was statistically significant between the pre-introduction generation (1988–1993 birth cohort) and the vaccination generation (1994–1999 birth cohort) (58.4% [90/154] vs. 40.0% [18/45], *p* = 0.04). Additionally, the HPV16/18 prevalence in CIN2–3/AIS was only 5.9% among vaccinated women aged 20–24 years (*n* = 51) in the 1988–1999 birth cohorts; we confirmed that attribution of HPV16 and HPV18 to CIN2-3/AIS differed remarkably between vaccinated and unvaccinated women (5.9% [3/51] vs. 59.3% [118/199], *p* = 0.00001).

These results demonstrated that HPV16/18 prevalence in CIN1 and CIN2–3/AIS diagnosed at age 20–24 years in unvaccinated women was significantly reduced in the 1994–1999 birth cohorts as compared with the 1988–1993 birth cohorts over the 9-year study period. For HPV31/33/45 and HPV52/58, however, no significant increase or decrease in prevalence was observed (Figure 2).

## 4. Discussion

We previously reported the effectiveness of HPV vaccination by comparing HPV16/18 prevalence in CIN1 and CIN2–3/AIS between vaccinated and unvaccinated women in Japan [15,16]. Apart from direct protection among vaccinated women, the present study is focused on changes in the HPV16/18 prevalence among unvaccinated women. We demonstrated a significant decline in the HPV16/18 prevalence among unvaccinated women aged 20–24 years with CIN1 during a 9-year period after introduction of the Japanese HPV vaccination program, with a similar time trend observed for CIN2–3/AIS. Among unvaccinated women, attributions of HPV16 and HPV18 to CIN1 and CIN2–3/AIS diagnosed at age 20–24 years was significantly reduced in the vaccination generation (1994–1999 birth cohorts) compared with the pre-introduction generation (1988–1993 birth cohorts). Our findings strongly suggest the herd effects of HPV vaccination in Japan. To the best of our knowledge, this is the first observation to report the herd effects of HPV vaccination in Japan. In a girls-only vaccination program, herd effects among unvaccinated women are most likely derived from unvaccinated but indirectly protected, heterosexual men. A recent Dutch study reported decreasing trends in HPV16/18 prevalence among both women and heterosexual men after the introduction of a girls-only vaccination program [20]. Although we did not assess the HPV16/18 prevalence in young Japanese men, these observations suggest that HPV16/18 prevalence among young Japanese men may be decreasing.

Herd effects of HPV vaccination have been observed in other countries with high coverage and/or gender-neutral vaccination programs [10,11,12,13]. A recent meta-analysis demonstrated that multiple age-cohort vaccination and high vaccination coverage contribute to herd protection [10]. Furthermore, herd effects of HPV vaccination on unvaccinated women may require more time to be measured than direct protection among vaccinated women because herd effects are an indirect benefit attributable to reduced transmission of vaccine-type HPVs in the population. In the United States and the Netherlands, significant declines in HPV16/18 prevalence among young unvaccinated women were not yet observed within 6 years post vaccination but became measurable 8 years after the introduction of HPV vaccination [12,13]. In Japan, high vaccine coverage in the 1994–1999 birth cohorts and a long period since the introduction of the HPV vaccination program may also have enabled us to observe herd effects with prolonged monitoring in Japan.

It is important to analyze time trends in vaccine type-specific prevalence among both vaccinated and unvaccinated women to evaluate the full benefit of vaccination, including herd effects. A prospective cohort study of Japanese women born in fiscal years 1993–1996 in Niigata Prefecture reported a lower incidence of HPV16/18 infections among vaccinated women compared with unvaccinated women (0.2% vs. 2.2%, *p* < 0.01) [9]. Using this comparison among women approximately 20 years of age, Kudo et al. estimated a vaccination effectiveness of 93.9% against HPV16/18 infections, after adjusting for sexual activity and birth year. However, this vaccine impact may have been underestimated owing to herd effects as the HPV16/18 prevalence (2.2%) among unvaccinated women aged around 20 years in the Niigata study was much lower than that (9.7%) reported among Japanese healthy women aged 20 years in the pre-vaccination era [21]. Given the high vaccine coverage (74.6%) in the Niigata study, herd effects may have diminished the difference in HPV16/18 prevalence between vaccinated and unvaccinated women. Time-trend analyses of HPV prevalence among both vaccinated and unvaccinated women would be needed to avoid underestimating the true benefits of HPV vaccination.

In CIN1 and CIN2–3/AIS from unvaccinated women aged 20–24 years, HPV16/18 prevalence decreased significantly from the 1988–1990 birth cohort to the 1997–1999 birth cohort. The lowest prevalence of HPV16/18 in the 1997–1999 birth cohort may be associated with the highest vaccine coverage in the 1997–1998 birth cohort [1]. In the present study, herd effects among unvaccinated women were even observed in the pre-introduction generation; HPV16/18 prevalence in CIN1 was significantly lower in the 1991–1993 birth cohort than in the 1988–1990 birth cohort. Although the MINT study did not assess indirect herd effects among men, this difference might have resulted from the difference in opportunities to have sexual intercourse with young men indirectly protected against HPV16/18 infections.

The present study has several limitations. First, we classified women as unvaccinated if they reported that they had not been vaccinated; the vaccination status of study participants was not validated against official vaccination registries. Possible misclassification of vaccination status might have affected the findings regarding herd effects. Although a serological study showed a good correlation between self-reported HPV vaccination status and antibody levels [22], serum samples were not available to evaluate the accuracy of self-reported vaccination status in the current study. In studies verifying self-reported HPV vaccinations using vaccine registers, the accuracy of self-reports of not being vaccinated varied considerably among studies; 54.5% in a Japanese study [23] but 90.0% in an Australian study [11] and 92.5% in a study from the United States [24]. In the present study, the HPV16/18 prevalence in CIN1diagnosed at age 20–24 years was very low (4.5%, 1/22) in the 1994–1999 birth cohort. Even if we assume that the accuracy of self-reports was 60% (i.e., 40% incorrectly classified as unvaccinated), the difference in HPV16/18 prevalence between 1988–1990 and 1994–1999 birth cohorts remained statistically significant for CIN1 (39.4% [13/33] vs. 7.1% [1/14], *p* = 0.04). On the other hand, Japanese municipal registries are not completely accurate because 1) vaccination records are not transferred when female adolescents move to another city after routine HPV vaccination, and 2) catch-up vaccination in female individuals aged >16 years is not recorded in the Japanese municipal registries. In some women, therefore, self-reporting may be better than official vaccination registries. Second, the HPV typing methods differed between 2012–2017 (LA) and 2019–2020 (PGMY-CHUV). Changes in laboratory methods might have affected the time trends of HPV16/18 prevalence. LA has been widely used in studies of HPV epidemiology, cervical cancer screening and vaccine surveillance, but was discontinued in December 2019. In the MINT study II, therefore, we selected PGMY-CHUV as an alternative HPV genotyping method. In our previous study comparing HPV genotyping results using both methods, the results with PGMY-CHUV were in complete agreement with those using LA for detection of HPV6, HPV11, HPV16, HPV18, HPV33, and HPV45 and showed near-complete agreement for HPV31 and HPV58 (98% and 99%, respectively) [19]. From these results, we consider that both assays are comparable for monitoring the impact of the bivalent and quadrivalent HPV16/18 vaccines. We could not confirm the HPV typing results of all patients by PGMY-CHUV because DNA samples collected in the MINT study I were not left available. Third, we are unable to exclude the possibility of confounding factors in time-trends and birth cohort analyses, such as changes in sexual behaviors, uptake of oral contraceptives, condom use, and the smoking rate. Educational programs and medical information after the introduction of the HPV vaccination program may have changed Japanese women’s understanding and behavior toward cervical cancer prevention. However, these changes are less likely to affect rates of HPV16/18 detected from cervical lesions than incidence rates of cervical lesions. Finally, the small sample size may have resulted in chance findings and limited precision for some analyses. To reduce the risk of chance findings, we analyzed changes in HPV16/18 prevalence for time trends and according to birth cohort separately.

## 5. Conclusions

In the present study, we found significant reductions in attribution of HPV16/18 to CIN1 and CIN2–3/AIS among unvaccinated women in the vaccination generation (1994–1999 birth cohorts), strongly suggesting the herd effects of HPV vaccination in Japan. To date, the MINT study has demonstrated vaccine type-specific evidence of direct and herd protection of the HPV vaccination program in Japan in previous [17] and current analyses, respectively. Continued monitoring over time in the MINT study will provide further valuable information to evaluate the protective effects of 9-valent HPV vaccines in addition to bivalent and quadrivalent HPV vaccines and effectiveness of catch-up vaccination in young women who missed routine vaccination owing to the government’s suspension of its vaccine recommendation.

## Figures and Tables

**Table 1:** Characteristics of unvaccinated cohorts.

	CIN1	CIN2–3 or AIS
	(*N* = 754)	(*N* = 4297)
**Registration Year**		
2012	58	172
2013	98	586
2014	94	602
2015	98	599
2016	95	642
2017	100	612
2019	21	78
2020	125	638
2021	65	368
**Age at Registration (Years)**		
20–24	95	210
25–29	199	923
30–34	251	1596
35–39	209	1568
**Birth Cohort**		
1973–1975	43	266
1976–1978	83	636
1979–1981	114	800
1982–1984	139	971
1985–1987	133	732
1988–1990	114	514
1991–1993	97	287
1994–1996	17	70
1997–1999	11	10
2000–	3	11
**HPV Genotypes**		
Oncogenic *	527	3932
HPV16	101	1753
HPV18	42	323
Non-oncogenic	118	159
Negative	109	206

HPV, human papillomavirus; CIN, cervical intraepithelial neoplasia; AIS, adenocarcinoma in situ; * Oncogenic HPV types include HPV16, 18, 31, 33, 35, 39, 45, 51, 52, 56, 58, 59, and 68.

## Data Availability

Not applicable.
